# Transforming technical assistance for more effective health services delivery in Africa: the WHO multi-country assignment teams experience

**DOI:** 10.3389/fpubh.2025.1560361

**Published:** 2025-06-18

**Authors:** Ndoungou Salla Ba, Abdulmumini Usman, Alex Gasasira, Patrick Kabore, Bei Achu, Hyelni Kulausa, Emmanuel Chanda, Olushayo Oluseun Olu, Joseph Cabore, Matshidiso Moeti

**Affiliations:** World Health Organization African Regional Office, Brazzaville, Republic of Congo

**Keywords:** technical assistance, multi country assignment teams, World Health Organization, WHO African region, WHO African regional office, Africa

## Abstract

The importance of technical assistance in fast tracking countries’ progress to the attainment of global and regional public health goals cannot be overemphasized. In this regard, the World Health Organization Regional Office for Africa, piloted a new, innovative, and stepwise technical assistance delivery approach from 2022 called the Multi-Country Assignment Teams. Given the experimental nature of this approach, this case study assessed its effectiveness in enhancing the quality and timeliness of technical assistance in the region, as well as identifying and addressing any initial challenges. The progress of MCAT activities were assessed using secondary data sourced from the MCAT monitoring and evaluation database, along with travel data from WHO’s Global Management System (GSM) covering April 2022 to September 2023. The findings highlight the potential of the Multi-Country Assignment Teams as a promising solution for providing quality and timely technical assistance to African countries. Most (67%) of the 928 technical support activities planned between April 2022 to December 2023 were successfully implemented. In general, most Multi-Country Assignment Teams host countries received more support than non-host countries except for Mozambique, Zimbabwe, and Kenya Multi-Country Assignment Teams where the technical support was greater in non-host countries. Data on the Multi-Country Assignment Teams duty travels revealed that there was a total of 185 missions in 2022. Out of this, 65 (35%) were within the Multi-Country Assignment Teams’ host country, 27 (15%) were in non-host countries, and 93 (50%) were not Multi-Country Assignment Team-related. Despite these achievements, several challenges that hinder the model’s accelerated implementation persist. These include delays in implementing the approach’s activities due to delayed recruitment of staff and inadequate funding and maldistribution of the approach’s activities between host and non-host countries. Additionally, many of the approach’s activities were non-related to its core function which hampered the effective and timely delivery of their support. Moving forward, it is crucial to build on the successes so far achieved by Multi-Country Assignment Teams while addressing the challenging issues, particularly by improving awareness of their functions and ensuring adequate staffing and funding.

## Introduction

Technical Assistance (TA) is the process by which an expert provides the knowledge, skills, and competencies to meet an individual’s or organization’s goals. Effective TA should be collaborative, systematic, targeted, flexible, and result oriented (1). Well-planned and delivered TA could result in stronger, more resilient health systems, and ultimately contribute to improved health outcomes and well-being for all (2–4). The critical role of rapid, tailored TA in achieving public health programmatic aims and objectives cannot be overemphasized. This became more evident during the unprecedented epidemic of Ebola in West Africa in 2014–2016 and the coronavirus (COVID-19) pandemic (5) when African national health authorities were overwhelmed by the outbreaks and sought support to effectively and timely prepare for and respond to these outbreaks.

Furthermore, several African countries continue to struggle to achieve the health-related Sustainable Development Goals (SDGs) such as goals 2, 3, 5, 6, 10, 13 and 17 due to limited capacity, expertise, and resources (6, 7). Despite financial support from initiatives such as the Global Polio Eradication Initiative (GPEI), the President’s Emergency Plan for AIDS Relief (PEPFAR), the Global Alliance for Vaccines and Immunization (Gavi the Alliance), and the Global Fund to Fight AIDS, Tuberculosis, and Malaria (GFATM), these countries often rely on TA from United Nations (UN) organizations such as the World Health Organization (WHO) and international non-governmental organizations to enhance their abilities to meet regional and national health targets (8).

Against this backdrop and recognizing the need to enhance its capacity to support its member states with effective TA, the WHO Africa Regional Office (WHO/AFRO) launched a major organizational reform called the Transformation Agenda starting from 2015. As part of the reforms, WHO/AFRO conducted a Functional Review (FR) of the 47 WHO country offices (WCOs) in the WHO Africa Region (WHO/AFR) from 2017 to 2019. The objectives of the FR were to ensure that the WCOs were fit-for-purpose and receive cutting edge TA from the WHO/AFRO to implement national and regional health strategies and programmes. Feedback from stakeholders such as the national health authorities, UN system partners, non-governmental organizations, and bilateral partners that were consulted during the FR highlighted the need for WCOs to provide timelier and better-quality TA. Specifically, the partners sought among others for better quality TA to implement their health priorities, coordinate the health partners, provide health information for evidence-based decision making and better integration of TA. Additionally, the lessons learned from the FR demonstrated the need for a paradigm shift to ensure that the work of WHO/AFRO and its WCOs is aligned with country priorities and that the best TA delivery modality is used (9–11).

Hitherto, WHO/AFRO provided TA to its member states through three Inter-Country Support Teams (ISTs) based in Burkina Faso for West Africa, Gabon for Central Africa, and Zimbabwe for Eastern and Southern Africa. However, this strategy faced significant challenges, including ad-hoc, vertical, short-term TA that was often delayed and limited capacity to meet the growing demand for TA across a diverse and geographically expansive region of 47 countries. Additionally, political, immigration, and bureaucratic barriers, along with insufficient funding, further complicated the delivery of TA. The three IST locations were far from most of the countries that they were assigned to support, which does not align with the WHO’s country-focused approach. Additionally, the IST system did not have a formal monitoring and evaluation system which could be effectively used to monitor its progress and achievements. To address these challenges, WHO/AFRO piloted a new, innovative, and stepwise TA delivery strategy called the Multi-Country Assignment Teams (MCATs) starting from 2022. Such new strategies require formative evaluation of their achievements and challenges to guide effective implementation. To the best of our knowledge, there are no studies that have done this.

Therefore, using the early information generated from the MCAT’s monitoring and evaluation system, we reviewed and evaluated whether the strategy is achieving its goals of enhancing TA quality with a view to identifying and addressing the initial challenges (12). This is in line with the best global practices in assessing the effectiveness of interventions that are geared toward improving organizational cohesiveness and functionality (13). To assess the progress of MCAT activities, secondary data were sourced from the MCAT monitoring and evaluation database, along with travel data from WHO’s Global Management System (GSM) covering April 2022 to September 2023. The data were consolidated in an Excel-based master database and analyzed using Excel formulas to track key indicators, including the overall and disaggregated progress by location and programme area, type of technical support, and equity in support distribution between host and non-host countries. Results were presented using various chart types, and qualitative information from programme reports were summarized to highlight common challenges and lessons learned.

## The context: the WHO Africa region and its public health status

WHO is the specialized health agency of the UN, divided into six regions, namely Africa (WHO/AFR), the Americas (PAHO), Eastern Mediterranean (EMRO), Europe (EURO), Southeast Asia (SEARO), and Western Pacific (WPRO). WHO/AFR consists of 47 member states^1^ that are primarily located in sub-Saharan Africa and is governed by a Regional Committee made up of Health Ministers from these states. The secretariat, known as WHO/AFRO, carries out the organization’s six core functions in the region, which include providing regional leadership in public health, fostering collaborative partnerships, shaping research agendas, establishing, and monitoring norms and standards, formulating ethical and evidence-based policies, and offering technical support for national health priorities while monitoring public health trends.

WHO/AFR member states face significant public health challenges compared to other WHO regions. With less than six years remaining to achieve global and regional development goals such as the SDGs, the Sendai Framework for Disaster Risk Reduction, and the African Union Agenda 2063, the region is currently off track. For instance, the Universal Health Coverage (UHC) service coverage index in the region is estimated at 48.71, significantly lagging other regions (14). There are stark disparities within the continent, with many low-income countries falling below the continental average (14). As of 2023, the coverage rate of fully immunized children stands at about 70%, which is insufficient to meet targets for eradicating vaccine-preventable diseases (15). Additionally, maternal and child mortality rates are far below what is required to achieve the SDGs (16).

Communicable diseases like HIV/AIDS, malaria, and tuberculosis continue to be prevalent, compounded by a growing burden of non-communicable diseases, resulting in a dual disease burden (17). Recurrent outbreaks of diseases such as viral hemorrhagic fevers, yellow fever, Mpox, cholera, measles, and poliomyelitis further strain the health systems of African countries (18). These challenges underscore the urgent need for effective, timely, and high-quality TA to support public health planning, implementation, monitoring, and evaluation in the region.

## The multi country assignment teams: definition, roles and current programmatic elements

MCATs are small teams of five to six health experts who provide technical oversights to a group of three or four WCOs based on geographical proximity, public health similarities, and language considerations (Table 1). Large countries such as the Democratic Republic of Congo, Ethiopia, and Nigeria, which require continuous high-level expert health support due to their significant health challenges, budgets and complexities, are excluded from the MCAT arrangements, as are countries in emergency situations like the Central African Republic and South Sudan. The Republic of Congo is also excluded due to a special memorandum of understanding with WHO/AFRO to provide direct TA to the country.

**Table 1:** List of MCATs, their location, coverage, technical capacity and support functions and achievements.

MCAT host country	# of experts	Portfolio/ Coverage	Current technical Capacity/ Functions	# (%) of planned activities	Activities I progress or completed	Activities not started
Burkina Faso	5	Burkina Faso, Niger, Togo and Benin	HIV/TB, NCDs, Nutrition, RMNCH, TVD	139 (15%)	91 (65%)	48 (35%)
Cote d’Ivoire	4	Cote d’Ivoire, Guinea and Mali	Health Financing, HIV/TB, RMNCH, TVD	134 (14%)	108 (81%)	26 (19%)
Gabon	3	Gabon, Chad, Cameroon and Equatorial Guinea	Health Financing, RMNCH, TVD	102 (11%)	63 (62%)	39 (38%)
Ghana	3	Ghana, Sierra Leone, Liberia and Gambia	HIV/TB, RMNCH, TVD	56 (6%)	37 (66%)	19 (34%)
Kenya	4	Kenya, Seychelles, Mauritius and Rwanda	Health Financing, Nutrition, RMNCH, TVD	105 (11%)	47 (45%)	58 (55%)
Madagascar	4	Madagascar, Comoros and Burundi	Health Financing, HIV/TB, Nutrition, RMNCH	109 (12%)	68 (62%)	41 (38%)
Mozambique	2	Angola, Mozambique, Cap Verde, Sao Tome & Principe and Guinea-Bissau	NCDs, RMNCH	53 (6%)	35 (66%)	18 (34%)
Senegal	3	Senegal, Mauritania and Algeria	Health Financing, RMNCH, TVD	90 (10%)	79 (88%)	11 (12%)
South Africa	3	South Africa, Eswatini, Botswana and Lesotho	HIV/TB, RMNCH, TVD	26 (3%)	24 (92%)	2 (8%)
Uganda	2	Uganda, Tanzania and Eritrea	RMNCH, TVD	28 (3%)	17 (61%)	11 (39%)
Zimbabwe	4	Zimbabwe, Zambia, Malawi and Namibia	Health Financing, NCDs, Nutrition RMNCH	86 (9%)	52 (60%)	34 (40%)
Total	37	41 countries	-	928	621 (67%)	307 (33%)

The primary aim of the MCAT is to provide high-level, integrated public health TA to WHO/AFR member states in their drive toward the attainment of the health-related SDGs particularly food security, good health and wellbeing, water and sanitation and climate action. Additionally, the MCATs aims to support the adaptation of global and regional health development goals to national contexts, policies, and priorities to achieve these objectives. MCATs focus on key functional areas such as Reproductive, Maternal, Neonatal, Child, and Adolescent Health (RMNCAH), HIV/TB/Hepatitis (HTH), Tropical and Vector-Borne Diseases (TVD), Non-Communicable Diseases (NCD), Health Financing (HF), and Nutrition, along with crosscutting areas like Diagnostic and Laboratory Services (DLS) and Service Delivery Systems (DSD). Addressing these areas is crucial for reducing morbidity and mortality and achieving SDGs and UHC in Africa.

The MCATs’ key roles include helping countries to consolidate health data and information to track progress and identify challenges, ensuring timely and integrated public health support, fostering cross-border collaboration, and sharing best practices and impact case studies. Additionally, MCATs are expected to mainstream gender, human rights, equity, the social determinants of health, culture, climate change and multi-sectoral collaboration into their TA. Contrary to the previous TA mechanism of WHO/AFRO, a monitoring and evaluation tool was developed to assess the quality of technical support provided by the MCAT teams. This tool includes various indicators to track functional areas and country portfolios, types of support offered, progress in implementing planned activities, and fund utilization. It also measures the achievement of their outputs as documented in their work plan in relations to the broader organizational Thirteenth Global Programme of Work (GPW13).

**Figure 1:**
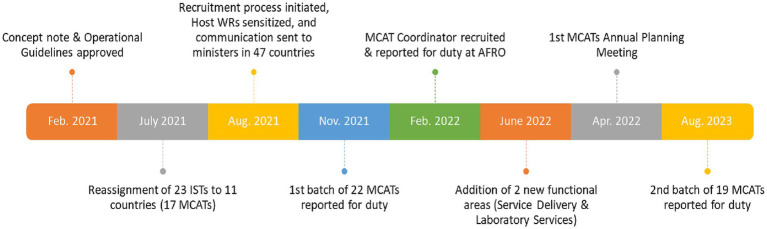
Timeline of MCATs establishment.

The MCAT concept note, and operational guidelines were approved and rolled out in February 2021 and the first batch of 22 MCAT members deployed to 11 countries in November 2021 (Figure 1). The second batch reported for duty in August 2023 (Figure 1). As of September 2024, 11 MCATs comprising 37 staff covering 41 countries in 6 out of 8 MCAT functional areas have been established (Table 1). Two functional areas, namely integrated services delivery and diagnostic and laboratory services are not yet functional in any of the MCATs. RMNCAH functional area has the highest representation in the MCAT with a presence in 10 out of 11 host countries, while the lowest is nutrition in 4 of 11 host countries (Table 1).

## Achievements of the multi country assignment teams

A total of 928 technical support activities involving 6 of 8 MCATs’ programme areas were planned between April 2022 to December 2023. Of the 11 locations, Burkina Faso had the highest number of activities (139) followed by Cote d’Ivoire 134, compared to South Africa, which had the lowest of 26. Three locations (Gabon, Cote d’Ivoire, and Madagascar), each planned more than 100 activities. Most of the activities (621, 67%) of the activities have either been successfully completed or are in progress while 307 (33) have not started (Table 1). Cote d’Ivoire, Senegal, and South Africa MCATs have more than 80% of planned activities in progress or successfully completed.

**Figure 2:**
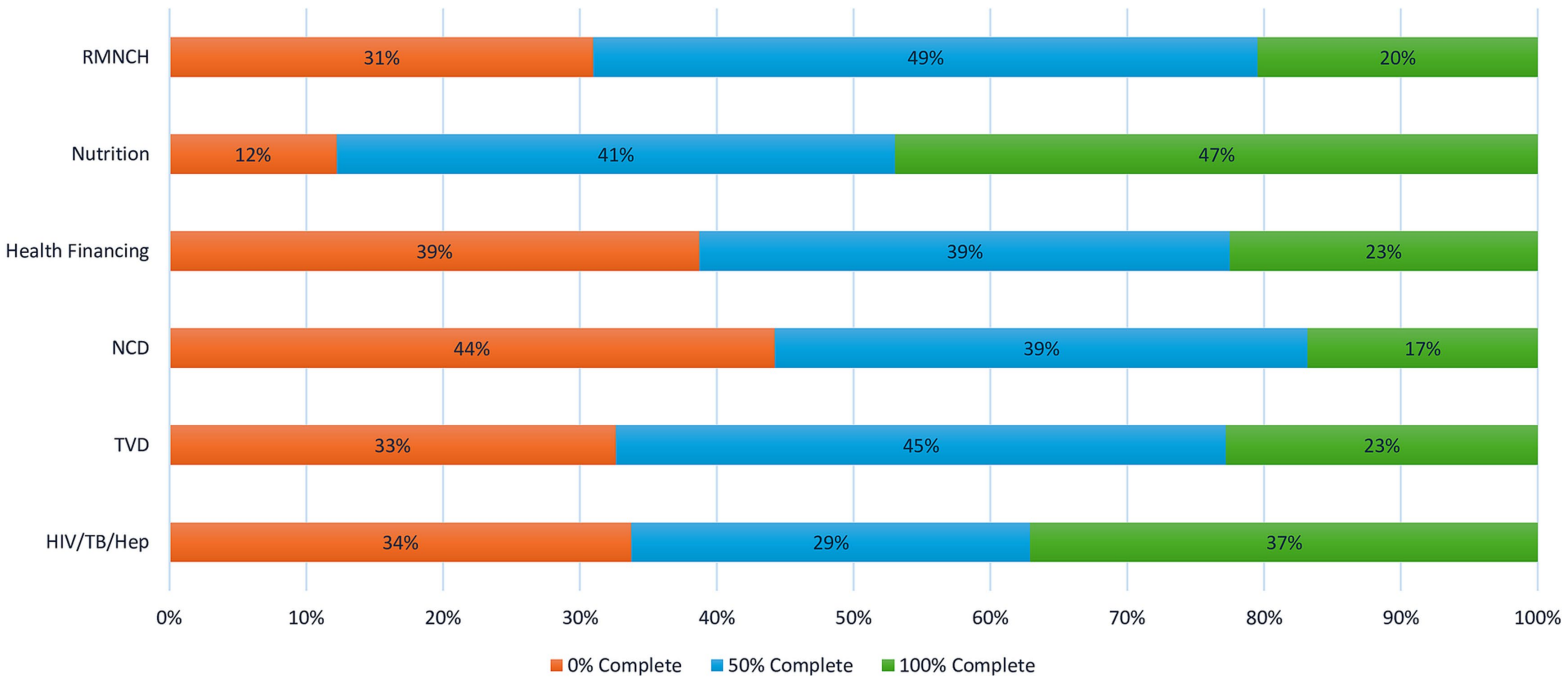
Achievements of MCATs by technical programme area.

In terms of achievements by technical programme areas, the HTH programme has the highest achievement of 37% of successfully completed activities followed by TVD and HF at 23% each and RMNCH at 20% (Figure 2). The NCD programme has the most uncompleted activities at 44% followed by health financings HIV/TB/Hep (34%) and TVD (33%) programmes (Figure 2). The successfully completed or in-progress activities are four key areas namely: (1) capacity building to enhance health system performance, (2) revision and elaboration of health strategic documents, policies, and frameworks across multiple countries, (3) cross-border collaboration to expand the reach of interventions and (4) coordination with Ministries of Health and partners engagement to mobilize resources.

In general, the majority of MCAT host countries received more support than non-host countries except for Mozambique, Zimbabwe, and Kenya MCATs where the technical support was greater in non-host countries. Most TA activities did not start in non-host MCAT such as Mali (37%) Equatorial Guinea (52%), Sierra Leone (47%), Kenya (73%), Comoros (52%), STP (44%), Algeria (23%), Botswana (33%), Eritrea and Tanzania (each 50%), Zambia (64%). Data on the MCATs duty travels revealed that there was a total of 185 missions in 2022. Out of this, 65 (35%) were within the MCAT’s host country, 27 (15%) were in non-host countries, and 93 (50%) were not MCAT-related.

## Discussion

This discussion focuses on the key issues, lessons learned and future directions for MCATs. The available MCAT data shows that it has made considerable progress in increasing the quantity as well as the quality and sustainability of WHO/AFRO’s TA to its member states. This is in line with the literature that has explored effects of organizational changes (19). The successful deployment, planning and implementation of a significant number of programme activities by the MCAT within a period of 18 months demonstrates its feasibility and capability to improve the quantity and timeliness of TA to the countries of the region. The fact that the MCAT location with the highest number of health experts implemented the highest number of activities underscores the importance of ensuring optimum staffing for the MCATs.

Nevertheless, the approach experienced a few challenges. First, the finding that several of the activities of the MCATs’ activities were not started or are uncompleted could be attributed to the long period that it takes to recruit and deploy teams. These delays placed additional pressure on existing resources, leading to an uneven distribution of TA across the region. Additionally, recruitment was compounded by inadequate funding. The financial constraints also hampered the execution of planned TAs, especially in non-host countries which left many activities either incomplete or not started. The lack of funding is generally due to a general shift in the development aid architecture in Africa from multilateralism to bilateralism (20). Second, as shown by the available evidence, many of the MCATs’ activities have been focused on the host countries at the detriment of the non-host countries such as Mali and Equatorial Guinea, experiencing significant delays or non-implementation of planned activities. Perhaps this is due to the proximity and convenience of providing TA to those in their immediate environment. However, this could also be attributed to the host countries monopolizing the MCAT’s support. Third, the fact that half of the MCATs duty travels were for non-MCAT related activities is a critical challenge which could hamper the effective and timely delivery of their support. This finding is largely due to a shortage of human resources in the WHO/AFRO thus MCATs staff members are often called upon to fill the gaps. Fourth, given that it is a recently implemented approach, there is inadequate understanding of its role among the staff members in host WCOs. In some instances, staff members view the MCATs as duplicating their roles resulting in anxiety and rejection, while in other instances they are well received and seen as additional capacities to implement the day-to-day activities of the office as opposed to the TA functions (21).

A few important lessons were learned from the implementation of the MCATs. Its modest success in delivering effective TA was largely dependent on the expertise and proximity of the teams involved to the countries that they support. Contrary to the previous TA provision system, the proximity of the MCATs not only aligns to the country-focused approach of WHO but has also facilitated easy access to the countries and eased travel logistics and bureaucracies. Experts with extensive international experience and deep knowledge of both global health issues and local contexts were instrumental in enhancing the capacity of the WCOs and Ministries of Health (MOHs). Thus, it is crucial to maintain high standards in selection of MCATs staff to sustain and improve their effectiveness (22). Additionally, the importance of coordination between the WHO Representatives and Country Directors (WRs) in both host and non-host countries emerges as a key lesson. Locations where WRs facilitated active dialogue and facilitated cross-country collaboration saw better outcomes, underscoring the need for continued joint coordination efforts.

Since it was found that joint coordination meetings and retreats played crucial roles in building team cohesion, streamlining priorities, and clarifying roles, strengthening such coordination will be key to future success. The ability of MCATs to leverage their expertise to mobilize additional resources was another key lesson. In cases where this was done successfully, the impact of limited funding was mitigated. Strengthening strategies to harness MCAT expertise for resource mobilization will be essential to ensure sustainable funding for the initiative. Furthermore, MCATs have proven highly effective in fostering cross-border collaboration, enabling countries to address shared public health challenges through joint projects. This approach not only strengthens regional cooperation but also expands the reach of interventions, yielding significant results.

Moving forward, several key areas of the MCATs activities require attention to further enhance the approach. Ensuring an equitable distribution of support between host and non-host countries is critical. Improved planning, resource allocation, and proactive engagement with non-host countries will help close the gaps in TA. Addressing recruitment and deployment delays is also vital. This can be achieved by streamlining recruitment processes and securing adequate funding from the outset. Additionally, more stringent assessment of candidates for MCAT positions would ensure that highly skilled experts are recruited. Clear communication of the roles of MCATs is through orientation sessions for both WCO and MCAT staff members will help ensure seamless integration of MCAT teams and the smooth implementation of their technical assistance mandates.

Additionally, reducing the number of non-MCAT-related duty travels will allow teams to focus on their core responsibilities, enhancing their effectiveness. This could be achieved by ensuring that WHO/AFRO have a full complement of staff and putting in place mechanisms that deter WHO/AFRO programme managers from assigning MCATs staff to non-MCAT related activities. Future efforts should also prioritize cross-country and cross-sectoral collaboration, particularly in underrepresented areas such as DSD and DLS. These sectors are key to advancing broader health system strengthening and achieving the health-related SDGs. By ensuring that all functional areas are operational, MCATs can provide more comprehensive and integrated support to member states. Finally, there is a need to ensure the sustainability of the MCAT system through the provision of predictable and flexible funding and its entrenchment into the organigrammes of WHO/AFRO and all WHO/AFR WCOs.

The findings and recommendations of this study should be interpreted in light of several limitations. First, all authors are affiliated with WHO, either as staff or consultants. While this provides valuable insight into the internal workings of the MCATs, it may introduce bias. Second, the predecessor to the MCATs, the ISTs, lacked a monitoring and evaluation system, leaving no quantitative data or indicators for comparison. Third, as the MCAT system is still in its early stages, assessing its impact remains challenging. Fourth, the findings are not generalizable; however, they offer useful insights that could help improve the MCATs and similar systems. These limitations were partially addressed by involving an independent consultant (HK) in the evaluation and including authors not directly engaged in MCAT implementation. We have also ensured that both the achievements and challenges of the MCATs were presented and discussed. A more comprehensive mid-term evaluation is required to address these limitations more thoroughly.

## Conclusion

WHO/AFR Member States are still falling short in achieving global and regional development and humanitarian goals, hindered by weak health systems and recurring infectious disease epidemics. Unfortunately, the TA delivery models from regional public health organizations are suboptimal, prompting WHO/AFRO to develop the MCAT model. Preliminary data from this case study highlight the potential of the MCATs as a promising solution for providing quality and timely TA to African countries. However, several challenges persist that hinder the model’s accelerated implementation. The findings provide useful insights which could guide future interventions and initiatives aimed at strengthening health systems in Africa and TA effectiveness toward the attainment of regional and global health development goals in the region. Nevertheless, the findings should be interpreted within the context of limitations such as the authors’ affiliation with WHO, lack of baseline data from the the previous system for comparison, early-stage implementation, and limited generalizability.

Moving forward, it is crucial to build on the successes so far achieved by the MCATs while addressing the challenges, particularly by improving awareness of their functions and ensuring adequate staffing and funding. Additionally, future research should focus on more quantitative evaluation of the outcomes and impacts of the MCAT system on the public health outcomes of the African member states. Furthermore, implementation research is required to define effective modalities for the implementation of all the aspects of the MCATs’ work.

## Data Availability

The original contributions presented in the study are included in the article/supplementary material, further inquiries can be directed to the corresponding author.

## Footnotes

^1^Algeria, Angola, Benin, Botswana, Burkina Faso, Burundi, Cameroon, Cape Verde, Central African Republic, Chad, Comoros, Ivory Coast, Democratic Republic of the Congo, Equatorial Guinea, Eritrea, Ethiopia, Gabon, Gambia, Ghana, Guinea, Guinea-Bissau, Kenya, Lesotho, Liberia, Madagascar, Malawi, Mali, Mauritania, Mauritius, Mozambique, Namibia, Niger, Nigeria, Republic of the Congo, Rwanda, São Tomé and Príncipe, Senegal, Seychelles, Sierra Leone, South Africa, South Sudan, Eswatini, Togo, Uganda, Tanzania, Zambia, Zimbabwe.
